# An intra-abdominal abscess or “rind” as a consequence of peritoneal dialysis-associated pseudomonas peritonitis

**DOI:** 10.5414/CNCS107951

**Published:** 2013-02-26

**Authors:** R. Michael Culpepper, Sarah Gore, Gregory W. Rutecki

**Affiliations:** 1 Division of Nephrology and Hypertension, and; 2 Resident in Radiology, University of South Alabama, Mobile, AL, USA

**Keywords:** peritoneal dialysis, peritonitis, Pseudomonas aeruginosa, rind formation

## Abstract

Background: Abdominal CT imaging has defined characteristics of two pathological entities specific to peritoneal dialysis patients. Both are associated with serious peritoneal complications. One is comprised of ascites accompanied by septation and loculated fluid pockets as a complication of bacterial peritonitis. The other is the syndrome of encapsulating peritoneal sclerosis. We present the evolution of a single, thick-walled fluid collection as a consequence of relapsing *Pseudomonas aeruginosa* peritonitis. The entity had distinctive features differing from either of the two previously described entities, and to our knowledge, has not been described previously. Our patient’s radiological evolution resembled the formation of a pleural or peritoneal “rind.” Conclusion: Peritonitis, as a result of *Pseudomonas aeruginosa*, may lead to “rind” formation as described with empyemas and is distinct from previously described intra-abdominal pathologies in peritoneal dialysis patients.

## Introduction 

Peritonitis is the most frequent cause of peritoneal dialysis failure [1]. While the overall incidence of peritonitis has decreased over the last two decades, the proportion of infections due to gram-negative organisms has increased [2]. Furthermore, when peritonitis is consequent to *Pseudomonas aeruginosa*, a serious gram-negative infection, it is less likely to be cured solely with antibiotics and more frequently leads to catheter removal as compared to other etiologies for peritonitis [1, 3]. The complicated course suggests severe inflammatory characteristics are present with pseudomonas peritoneal infections. 

Two distinctive complications of peritoneal dialysis result in increased morbidity and mortality. The first is the post-peritonitis development of ascites comprised of loculated fluid pockets [4, 5]. The second is the syndrome of encapsulating peritoneal sclerosis [6]. The former has been reported in 2 – 20% of patients requiring removal of peritoneal catheters consequent to peritoneal infections. 50% of these individuals also require percutaneous catheter drainage of persisting fluid, which is usually culture negative [5]. The latter syndrome has a mortality rate that may be as high as 50% and is associated with increased duration of peritoneal dialysis, but has not been correlated with the frequency or severity of peritonitis episodes [7]. 

Abdominal CT imaging has defined distinctive radiographic characteristics consistent with both entities. Septation and loculation of peritoneal fluid is virtually universal in the former and thickening of the peritoneal membrane and bowel wall with entrapment exemplary of the latter. Herein, we present the evolution of a large, single, persistent intra-abdominal fluid collection following episodes of relapsing *Pseudomonas aeruginosa* peritonitis. This fluid collection was unique, that is, different than either post-peritonitis fluid pockets or encapsulating peritoneal sclerosis. It lacked typical septations and loculations seen with post-peritonitis ascites, and although it contained a thick wall typical of peritoneal sclerosis, no other features characteristic of this syndrome were identified. Its particular appearance and evolution were analogous to previous descriptions of pleural and peritoneal “rinds” as result of infections or cancer. 

## Case report 

A 54-year-old man with biopsy-proven IgA nephropathy on peritoneal dialysis (PD) presented with a complaint of abdominal pain similar to previous episodes of peritonitis. Consequent to persistent emesis and volume depletion, he was admitted to the hospital. He had had outpatient treatment for four episodes of peritonitis over the past 5 years; the last, culture positive for *Citrobacter koseri*, 18 months prior to admission. 

He initiated PD in June 1997, but received a kidney transplant with a donor kidney from his brother 6 months later. In January 2005, he returned to PD due to chronic rejection and failure of his transplant kidney. After 3 years on PD, he underwent bilateral nephrectomy in March 2008, for persisting nausea and bilateral flank pain. A clear cell renal carcinoma (Stage T1, N0, Mx) was discovered in the right kidney and the left kidney had multicystic changes. Post-operatively, he was treated with hemodialysis via a tunneled dialysis catheter for 8 months when a peritoneal catheter was reinserted. He returned to PD in January 2009. 

**Figure 1.**
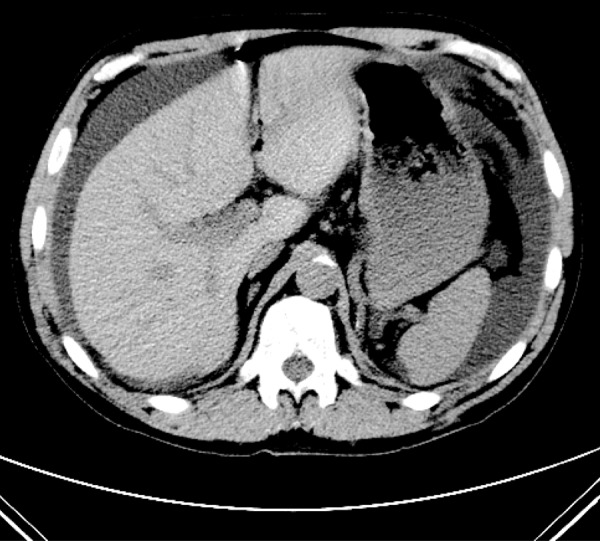
Axial, noncontrasted CT image at the level of the porta hepatis demonstrates freely mobile peritoneal fluid tracking along the liver, spleen, and left lateral abdominal wall at the initial diagnosis of pseudomonas peritonitis.

In July 2011, the index admission, his temperature was 96.7° with a peripheral white blood cell count (WBC) of 15,700/mm^3^ (normal differential count) and PD effluent cell count of 5,680/mm^3^ (a majority of neutrophils) (Table 1). A CT scan of the abdomen (Figure 1) demonstrated freely communicating intra-abdominal fluid and initial fluid samples grew *Pseudomonas aeruginosa*. Initial and subsequent therapy were not accomplished according to guidelines [8]. The patient was treated only with intra-peritoneal tobramycin (0.6 mg/kg/d), but experienced rapid resolution of his pain, clearing of fluid leukocytosis, and he was discharged on Day 4 to continue daily instillation of tobramycin. 

Recurrence of abdominal pain and nausea led to repeat hospitalization 2 weeks later with fluid again positive for *Pseudomonas aeruginosa*. Despite continuation of intra-peritoneal tobramycin and addition of oral levofloxacin, persisting symptoms led to removal of the PD catheter after 4 days (Table 1). A repeat CT scan of the abdomen again showed freely communicating intra-abdominal fluid. He improved on hemodialysis with continued intravenous ceftazadime and oral levofloxacin for 4 weeks. 

**Figure 2.**
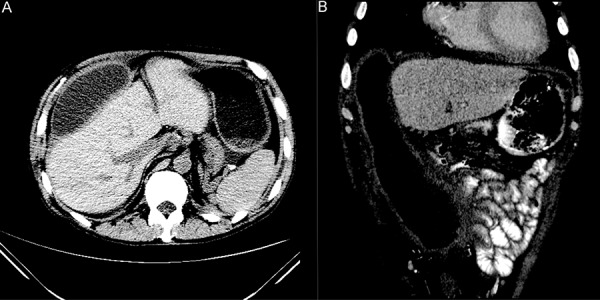
A: Axial CT noncontrasted image at the level of the porta hepatis obtained 18 weeks after index admission. There has been interval development of a loculated, thick walled fluid collection anterior to the liver. The perisplenic and perigastric fluid is no longer present. B: Coronal reconstructed image through the anterior abdomen at 18 weeks demonstrates the craniocaudal extent of the encapsulated fluid collection which extends from the right subphrenic space into the pelvis. Although the collection closely approximates and exerts a mass effect on adjacent small bowel, there is no invasion or trapping of bowel.

After 15 weeks, he had another episode of abdominal pain (Table 1) and at that time (Figure 2), CT scan of the abdomen demonstrated compartmentalization of abdominal fluid with the development of a thick wall or “rind” encapsulating a large right abdominal fluid collection. After 2 CT-guided drainage procedures and a 6-week course of parenteral ceftazadime and oral levofloxacin, he had complete resolution of symptoms. 

**Figure 3.**
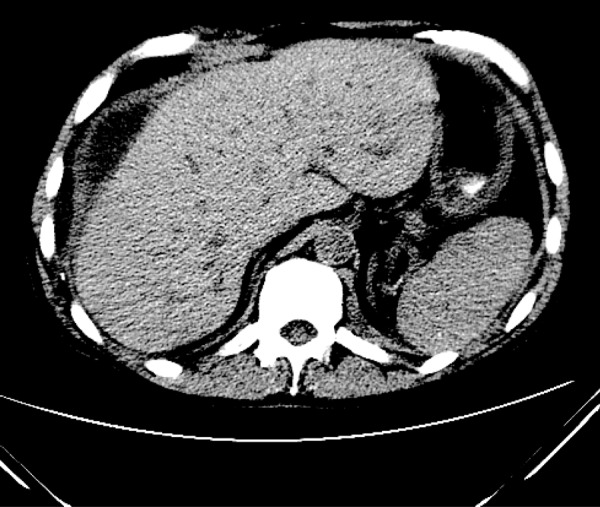
Axial, noncontrasted CT image slightly superior to the porta hepatis obtained at 8 months. The image demonstrates a persistent, encapsulated anterior perihepatic fluid collection (Culture negative). Although the collection has decreased in size from the previous exam, the “rind” is unchanged.

**Figure 4.**
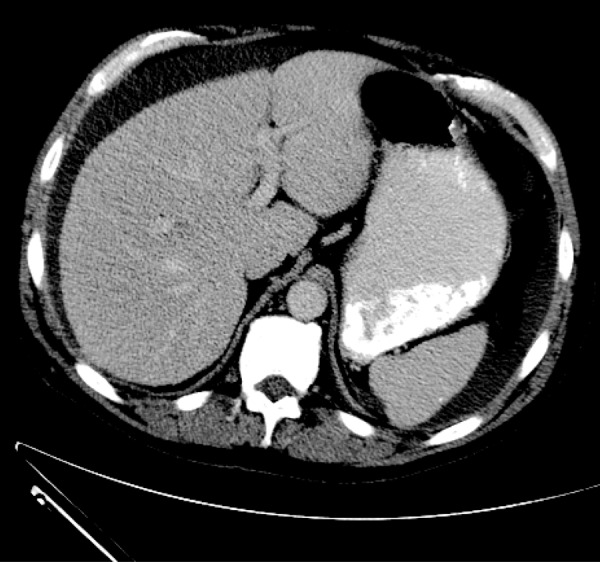
Axial noncontrasted image at the level of the porta hepatis obtained at 9.5 months. It demonstrates free-flowing, homogenous fluid tracking along liver, spleen, and left lateral abdominal wall. The “rind” has resolved.

Three months later, a CT scan was performed and revealed persistence of a large peri-hepatic fluid collection enclosed within the same thick wall (Figure 3). Fluid obtained via percutaneous drainage of the cavity was sterile (Table 1). A final CT examination of the abdomen 6 weeks later (Figure 4) showed continued diminution of the fluid collection, which again was sterile. Further problems have not recurred over 12 subsequent months. 

## Discussion 

*Pseudomonas aeruginosa* peritonitis is a complex and serious infection. Peritoneal dialysis-associated peritonitis due to this organism is less likely to respond to intra-peritoneal antibiotic administration and more likely to require removal of the peritoneal dialysis catheter in comparison to infections with other organisms [1, 3]. Current recommendations for initial treatment include two antibiotics, at least one administered intra-peritoneal, until culture results are available [8]. If a clinical response is not obtained in 4 days, which is defined as a reduction in the peritoneal effluent cell count, the guidelines recommend removal of the peritoneal catheter [8]. 

This patient’s complicated course may have been a result of inappropriate initial therapy and a lack of combination anti-pseudomonal treatment. While he was initially treated with only 1 of the 3 antibiotics to which his organism was sensitive, he had a rapid clinical response and reduction in the peritoneal effluent cell count. Despite this initial, positive response, he returned within 2 weeks with recurrent symptoms, increased fluid cell count, and repeat culture positive for *Pseudomonas aeruginosa*. At that time, addition of a second antibiotic failed to effect either a clinical or fluid cell count response after 4 days and the catheter was removed. 

Subsequent developments in this patient were atypical, especially when compared to previously reported pseudomonal peritoneal infections [1, 3, 4, 5] First, a large fluid filled cavity characterized by a thick “rind” restricted the fluid to a single demarcated area unlike the radiological findings described with ascites, septation, and loculation after bacterial peritonitis (Figure 2). Therefore, this picture differs from fluid compartmentalization associated with *Pseudomonas* or other bacterial etiologies, which are comprised of multiple septae. 

Second, the thickened wall in our patient was analogous to the syndrome of encapsulating peritoneal sclerosis (EPS). However, in contrast, EPS is not a result of peritonitis, but rather a consequence of prolonged peritoneal dialysis [7, 9]. Furthermore, bowel obstruction/entrapment, the cardinal manifestations of encapsulating peritoneal sclerosis, were absent in our case, and the sclerotic surface was confined only to the bowel wall. There was no evidence for symptomatic entrapment. 

Is there precedent for the unusual aspects of this patient’s course? Although this is the first report of this specific phenomenon complicating peritoneal dialysis to our knowledge, there are other situations characterized by a similar pathology. The literature has dubbed the inflammatory or malignant “walling off” pathology as rind formation. Furthermore, rind has been documented in both pleural and peritoneal cavities, usually the result of infection with a highly pathogenic organism or an infiltrating malignancy. The former occurrence is exemplified by infiltrative pleural pathology associated with a tuberculous or other bacterial empyemas (*Pseudomonas* or *Streptococcal pneumoniae*) [10]. The latter is either a pleural or peritoneal-based rind complicating a mesothelioma in the pleural cavity or a gastrointestinal adenocarcinoma or ovarian cancer in the peritoneal cavity [11]. There have been other appropriations of the term “rind” for sundry ultrasonographic phenomena, but these entities are not pathologically similar to the aforementioned pleural and peritoneal processes [12, 13]. 

In the setting of pleural and peritoneal rind formation, each is the result of various mediators stimulating an aggressive fibrinopurulent process. The intense inflammation culminates in septation and stranding all taking place within a dense, fibrous surrounding wall. After the inflammatory process is arrested, fibroblasts form a thick “peel,” walling off infectious, malignant, and/or sterile necrotic contents within. The most studied examples of rind formation are those resulting from empyema, especially in children [14]. The organisms responsible are considered aggressive and include *Pseudomonas* species [14]. 

Although the processes responsible for rind formation are not completely delineated, a number of factors seem to contribute. When the rind is a consequence of infection, the severity of infection, including the number of organisms, their virulence, and the intensity of the host response seem to correlate. For example, with tuberculous empyemas and resulting pleural rinds, greater numbers of bacteria are associated with a greater intensity of inflammation [15]. Likewise these same risks have also been described with *Streptococcus pneumoniae* rinds. In fact, *S. pneumoniae* isolates associated with rinds are more frequently antibiotic resistant [16]. From that perspective, our patient had a highly active, relapsing pseudomonas peritoneal infection with elevated fluid cell counts over 5 months. The inflammation and duration of infection consequent to his illness were protracted. Second, *Pseudomonas,* like *Mycobacterium tuberculosis* and *Streptococcus pneumoniae*, is remarkably pathogenic because of its ability to stimulate myriad inflammatory mediators while resisting anti-infective measures [17, 18]. This durability has been ascribed to siderophores, synergistic cooperation with other organisms, flagellae, adhesive molecules, plasmids leading to antibiotic resistance, biofilm formation, and exotoxins like elastase. The intensity and consequent damage from *M. tuberculosis* pleural-based inflammation also correlate with another highly active mediator in the metalloproteinase family. It is capable of digesting a variety of tissue elements [19]. The first peritoneal rind described herein as a result of pseudomonas peritonitis was similar to prior publications describing rind formation, especially those associated with tuberculous and pseudomonal empyemas. 

Models that specifically culminate in EPS have been developed. They are driven by a so-called “Two Hit” approach [20]. For example, the first “Hit” could be contingent upon bioincompatible peritoneal dialysis solutions; the second, blood-induced adhesions. Analogous “Hits” have been comprised of transforming growth factor β (TGF-β1) [21], chemical irritants (chlorhexidine gluconate) [22], or peritoneal catheters per se [23]. Although this patient’s peritoneal pathology was different from EPS, it has become clear that multiple, disparate inflammatory processes contribute to peritoneal injury in dialysis patients. The categories involved (catheters, inflammatory byproducts, and prolonged irritation/inflammation) were all present in this patient. 

This case report, to our knowledge, is the first to associate a peritoneal rind with *Pseudomonas aeruginosa *peritonitis after a protracted inflammatory process. Although the exact mechanisms responsible for the unusual pathology are unproven, the duration of infection, the specific organism, and a destructive combination of inflammatory mediators contributed. The pattern is analogous to previously described rind formations with *Pseudomonas empyemas*. In addition to a multi-septated cavity as a consequence of this debilitating and dangerous infection, it appears that under certain circumstances, pseudomonas peritonitis can be associated with rind formation that must be instrumented for relief of infection. 

## Conflict of interest 

The authors report no conflicts of interest. 

**Table 1.** Serial representation of clinical course, peritoneal findings, and therapy over 9 months in a peritoneal dialysis patient with protracted pseudomonas peritonitis complicated by rind formation.

Date	Clinical presentation	Culture/sensitivity of peritoneal fluid effluents	Cell counts (/mm^3^) from peritoneal fluids
7/3/11	Peritonitis – abdominal pain T-96.7°	*Pseudomonas aeruginosa * Tobra, Levo, Ceftaz*	PD-5.68k; WBC-16.1k (Figure 1)
7/17/11	Peritonitis – pain/nausea/vomiting T-98.4° BP-186/90	*Pseudomonas aeruginosa * Tobra, Levo*	PD-4.23k; WBC-51.0k
7/21/11	PD Cath out		
11/7/11	Peritonitis Abscess drained – blood-tinged clear straw-colored fluid	*Pseudomonas aeruginosa *Tobra, Levo, Ceftaz	PD-1.83k; WBC-8.9k
11/23/11	Abdominal pain T-98.5° BP-215/110 abscess drained – blood-tinged straw-colored fluid	*Pseudomonas aeruginosa *Tobra, Levo, Ceftaz*	WBC-12.9k (Figure 2)
12/6/11	Right lower quadrant pain T-98.4°	*Pseudomonas aeruginosa *Tobra, Levo, Ceftaz	
2/29/12	Follow-up Peri-hepatic fluid drained – clear, light brown fluid	No growth 5 days	WBC-11.7k (Figure 3)
4/11/12	Follow-up Peri-hepatic fluid drained – clear, yellow fluid	No growth 5 days	PD-53; WBC-20.7k (Figure 4)

*Abbreviations for tobramycin, levofloxacin, & ceftazidime.
